# Development of an FBG Sensor Array for Multi-Impact Source Localization on CFRP Structures

**DOI:** 10.3390/s16101770

**Published:** 2016-10-24

**Authors:** Mingshun Jiang, Yaozhang Sai, Xiangyi Geng, Qingmei Sui, Xiaohui Liu, Lei Jia

**Affiliations:** School of Control Science and Engineering, Shandong University, Jinan 250061, China; saiyaozhang@163.com (Y.S.); sdugengxiangyi@163.com (X.G.); qmsui@sdu.edu.cn (Q.S.); sduliuxiaohui@163.com (X.L.); jialei@sdu.edu.cn (L.J.)

**Keywords:** fiber Bragg grating, multi-impact localization, MUSIC algorithm, Shannon wavelet transform

## Abstract

We proposed and studied an impact detection system based on a fiber Bragg grating (FBG) sensor array and multiple signal classification (MUSIC) algorithm to determine the location and the number of low velocity impacts on a carbon fiber-reinforced polymer (CFRP) plate. A FBG linear array, consisting of seven FBG sensors, was used for detecting the ultrasonic signals from impacts. The edge-filter method was employed for signal demodulation. Shannon wavelet transform was used to extract narrow band signals from the impacts. The Gerschgorin disc theorem was used for estimating the number of impacts. We used the MUSIC algorithm to obtain the coordinates of multi-impacts. The impact detection system was tested on a 500 mm × 500 mm × 1.5 mm CFRP plate. The results show that the maximum error and average error of the multi-impacts’ localization are 9.2 mm and 7.4 mm, respectively.

## 1. Introduction

Due to its many advantages such as high strength, light weight, and resistance to corrosive environment, carbon fiber-reinforced polymer (CFRP) has become a popular construction material among modern structures. However, CFRP is prone to damage from external impact loads [1,2] that can significantly degrade the life of composite structures [3,4]. Identification and localization of impacts are important for the safety operation and low-cost maintenance of structures based on CFRP. In practical applications, the structures are often impacted by foreign objects at multiple locations, leading to significant challenges to the identification and localization of these impacts. Multi-impact localization can be mainly used for the impact events detection of aircraft structures; impacts can cause many invisible damage to the carbon fiber, which can seriously reduce the reliability of the structure, and threaten the safety of aircraft and personnel. During the process of collision, many impacts caused by external object splashes may occur at the same time, so the study of multi-impact localization can achieve a more accurate and effective method of damage monitoring and has an important academic research value.

Because of their small size, light weight, immunity to electromagnetic interference, and potential capability to be embedded in the composite structure [5,6,7], fiber optic sensors are attractive for impact detection and have been extensively studied in the past. Kirkby et al. [8] applied triangulation technology to locate an impact source on composite panels by using fiber Bragg grating (FBG) sensors. Fu et al. [9] used the hyperbolic curves algorithm and four fiber optic sensors to achieve impact localization on a composite plate. When multiple impact events simultaneously occur, signals from different impact sources need to be distinguished in order to obtain their relative timing information. With the development of artificial intelligent technology, intelligent algorithms were employed for impact source localization. Riberiro et al. [10] respectively used FBG sensors and the neural network method to obtain the location of the impact source on composite structures. Lu et al. [11] proposed support vector regression to determine the impact source on a composite structure using FBGs. In theory, artificial intelligence technology can achieve the localization of multiple impact sources. However, it usually requires a large number of training samples, which makes the localization a time-consuming, inefficient, and difficult process.

In recent years, more attention has been paid to finding better methods for the localization of multi-impact sources and many methods have been developed including the Kalman filter [12], applied elastodynamics and wavelet analysis [13], and the time reversal focusing method [14]. In our previous works, we have used least squares support vector machine [15] and extreme learning machine [16] to localize a single impact on a CFRP plate. Here, we expanded our work and developed a new impact detection system based on a FBG sensor array and a new localization algorithm that can identify the number and location of multiple impacts with high precision.

## 2. Localization Algorithm 

### 2.1. Principle of Multiple Signal Classification Algorithm

**Figure 1:**
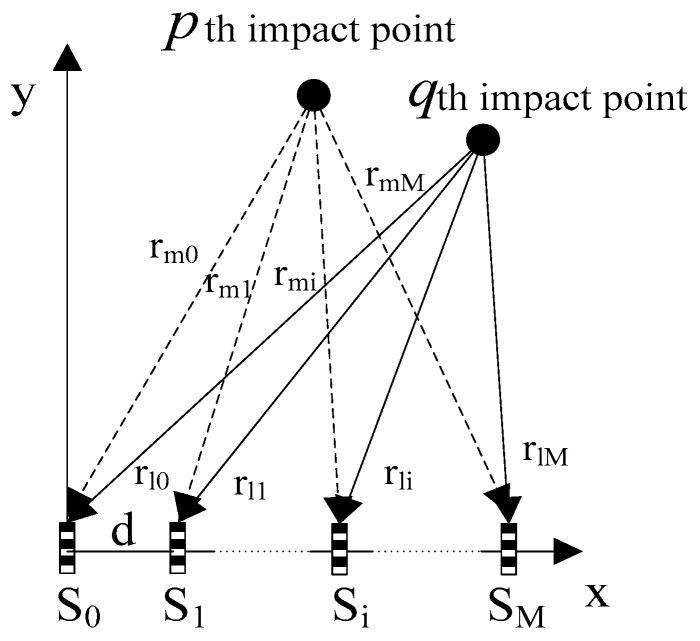
Localization algorithm.

The sensor array consists of *M* + 1 sensors that are equally spaced in a line. As shown in Figure 1, the spacing of adjacent sensors is *d* and the coordinate of a sensor (denoted as S_i_) is (*id*, 0) (*i* = 0, 1, … , *M*). Assuming that a number of *N* impacts occur simultaneously on the plate at locations of (*x_p_*, *y_p_*), where *p* = 1, 2, … , *N*, the signals from the impacts are detected by each of the sensors and the output of sensor S_i_ can be expressed as:

(1) $$z_{i}(t)=\sum_{p=1}^{N}s_{pi}(t)+n_{i}(t)$$

where *s_pi_* and *n_i_* are the signals detected by sensor S_i_ and the noise, respectively. The signals from impacts are elastic waves with a certain frequency component of *ω*. Using S_0_ as the reference sensor, Equation (1) can be rewritten as:

(2) $$z_{i}(t)=\sum_{p=1}^{N}s_{p0}(t)e^{-j\omega \tau_{pi}}+n_{i}(t)$$

According to the coordinates of the impact and sensors, the time difference ($\tau_{pi}$) is given by:

(3) $$\tau_{pi}=\frac{r_{pi}-r_{p0}}{c}=\frac{\sqrt{{\left(x_{p}-(id)\right)}^{2}+y_{p}^{2}}-\sqrt{x_{p}^{2}+y_{p}^{2}}}{c}$$

where *r_pi_* is the distance between an impact source and sensor S_i_, and *c* is the elastic wave velocity. Define **a***_p_*(*x_p_*, *y_p_*) as the position vector of the impact sources and it can be written as:

(4) $$a_{p}(x_{p},y_{p})=[1,\cdot \cdot \cdot ,e^{-j\omega \tau_{pM}}]$$

Using Equation (4), Equation (2) can be expressed in terms of vector operation or:

(5) Z(t)=A(x,y)S(t)+N(t)

where

$$\begin{array}{l} Z(t)={\left[z_{0}(t),\cdot \cdot \cdot ,z_{M}(t)\right]}^{T}\\ A(x,y)=[a_{1}(x_{1},y_{1}),\cdot \cdot \cdot ,a_{N}(x_{N},y_{N})]\\ S(t)=[s_{1}(t),\cdot \cdot \cdot ,s_{N}(t)]\\ N(t)={\left[n_{0}(t),\cdot \cdot \cdot ,n_{M}(t)\right]}^{T} \end{array}$$

Assuming that the signal and the noise are independent from each other and the noise can be considered as white Gaussian noise, the covariance matrix of the output signal vector can be expressed as:

(6) $$R=E[ZZ^{H}]=AE[SS^{H}]A^{H}+E[NN^{H}]=AR_{s}A^{H}+\sigma^{2}I$$

where **R***_s_* is the covariance matrix of the signal and *σ*^2^**I** is the covariance matrix of the noise.

Assuming the signal and noise are uncorrelated and the signal-to-noise ratio is high, the eigenvalue decomposition of **R** can be obtained by:

(7) $$R=U_{s}\sum_{s}U_{s}^{H}+U_{N}\sum_{N}U_{N}^{H}$$

where **U***_s_* is the signal subspace spanned by the eigenvector matrix corresponding to the greater eigenvalue, and **U***_N_* is the noise subspace spanned by the eigenvector matrix corresponding to the smaller eigenvalue.

Further assuming that signals from the *N* impact sources are independent from each other, the signal subspace **U***_s_* and the subspace spanned by the position vector are in the same space (*span*{**v**_1_, **v**_2_, … , **v***_N_*} = *span*{**a**_1_, **a**_2_,…, **a***_N_*}). At the same time, the signal subspace **U***_s_* and the noise subspace **U***_N_* are orthogonal (**A**^H^**v***_k_* = 0, *k* = *N* + 1, … , *M* + 1).

Because the ideal covariance matrix is unknown in practical applications, covariance matrix **R** is replaced by the maximum likelihood estimator $\hat{R}$ of **R** and $\hat{R}$ can be expressed as:

(8) $$\hat{R}=\frac{1}{L}ZZ^{H}$$

where *L* is the number of snapshots. To describe the orthogonal properties described above, the spatial spectrum is used by:

(9) $$P_{MUSIC}(x,y)=\frac{1}{a^{H}(x,y)U_{N}U_{N}a(x,y)}$$

According to Equation (9), the values of the spatial spectrum are calculated in the test area and the peak point of the spatial spectrum gives the location of the impact source.

### 2.2. Estimation of the Number of Impacts

According to Equation (7), the number of eigenvalues of the noise matrix need to be known. It is equal to the number of eigenvalues of the impact signal matrix, which is the same as the number of impacts.

The Gerschgorin disc theorem [17] is introduced for obtaining the number of impacts. In the M×M matrix $\hat{R}$, the sum of the magnitudes of *i*th row vector, which does not include the elements of *i*th column vector, can be expressed as:

(10) $$r_{i}=\underset{j\neq i}{\sum_{j=1}^{M}}\;\left|g_{ij}\right|\;i=1,2,\cdot \cdot \cdot ,M$$

The center and radius of the Gerschgorin disc are *g_ii_* and *r_i_*, respectively. All eigenvalues of $\hat{R}$ are contained in the union of the Gerschgorin discs. If a collection of *l* discs is isolated from the other discs, there exist exactly *l* eigenvalues of $\hat{R}$ in this collection. Before the Gerschgorin discs algorithm is used, $\hat{R}$ is rewritten as:

(11) $$\hat{R}=\left(\begin{array}{cc} \left(\begin{array}{ccc} R_{11} & \cdots & R_{1(M-1)}\\ \vdots & \ddots & \vdots \\ R_{(M-1)1} & \cdots & R_{\left(M-1)(M-1\right)} \end{array}\right) & \left(\begin{array}{c} R_{1M}\\ \vdots \\ R_{M(M-1)} \end{array}\right)\\ \left(\begin{array}{ccc} R_{M1} & \cdots & R_{M(M-1)} \end{array}\right) & R_{MM} \end{array}\right)=\left(\begin{array}{cc} R_{M-1} & r\\ r^{H} & r_{MM} \end{array}\right)$$

The matrix **R***_M_*_−1_ is decomposed as

(12) $$R_{M-1}=U_{M-1}\Lambda U_{M-1}^{H}$$

where Λ is the descending order diagonal matrix of the eigenvalues of **R***_M_*_−1_, and **U***_M_*_−1_ is the unitary matrix which is constructed by the corresponding eigenvector **e***_i_* (*i* = 1, 2, … , *M* − 1). According to **U***_M_*_−1_, a unitary transformation matrix **U***_T_* is obtained by:

(13) $$U_{T}=\left(\begin{array}{cc} U_{N-1} & 0\\ 0 & 1 \end{array}\right)$$

The matrix $\hat{R}$ can be transformed by:

(14) $$\begin{array}{ll} R_{T} & =U_{T}{}^{H}\hat{R}U_{T}=\left(\begin{array}{cc} U_{M-1}^{H}R_{M-1}U_{M-1} & U_{M-1}^{H}r\\ r^{H}U_{M-1} & r_{MM} \end{array}\right)\\ & =\left(\begin{array}{ccccc} \lambda_{1} & 0 & \cdots & 0 & \rho_{1}\\ 0 & \lambda_{2} & \cdots & 0 & \rho_{2}\\ \vdots & \vdots & \ddots & \vdots & \vdots \\ 0 & 0 & \cdots & \lambda_{M-1} & \rho_{M-1}\\ \rho_{1}^{*} & \rho_{2}^{*} & \cdots & \rho_{M-1}^{*} & r_{MM} \end{array}\right) \end{array}$$

where $\rho_{i}=e_{i}^{H}r$. The radius *r_i_* of the Gerschgorin discs, whose center is *λ_i_*, is expressed as:

(15) $$r_{i}=\left|\rho_{i}\right|=\left|e_{i}^{H}r\right|$$

All radiuses are ranked in descending order. According to the Gerschgorin disc theorem, the estimator of the impact number is defined as:

(16) $$\begin{array}{cc} GDE(k)=r_{k}-\frac{D(L)}{N-1}\sum_{i=1}^{M-1}r_{i} & k=1,2,\cdots ,M-2 \end{array}$$

where *L* is the number of snapshots, and *D*(*L*) is the adjustment factor. After all Gerschgorin discs estimator (GDE) coefficients are calculated, the number of positive GDE(k) gives the number of impacts.

### 2.3. Shannon Wavelet Transform

The multiple signal classification (MUSIC) algorithm described above requires the signal from the impact to be a narrow band. However, in practice, the signal from impacts usually is a short burst of elastic waves that has a wide band spectrum. The narrow band signal needs to be extracted for the application of MUSIC. Shannon wavelet transform is employed for extracting the narrow band signal from the output of the sensor [18].

The Shannon wavelet function is defined as:

(17) $$\psi_{m}(t)=\sqrt{f_{b}}\sin c(f_{b}t)e^{2\pi if_{c}t}$$

The Fourier transform of Equation (8) is:

(18) $$\Psi_{m}(\omega )=\{\begin{array}{cc} \sqrt{\frac{2\pi }{\omega_{b}}} & \omega_{c}-\frac{\omega_{b}}{2}<\omega \leq \omega_{c}+\frac{\omega_{b}}{2}\\ 0 & Others \end{array}$$

where $\omega_{b}=2\pi f_{b},\;\omega_{c}=2\pi f_{c},\;\omega_{c}>\omega_{b}/2,\;\Psi_{m}(\omega )$ is the rectangular window function. It can extract the narrow band signal whose center frequency is *ω_c_*. The bandwidth is limited in the range of ($\omega_{c}-\omega_{b}/2,\;\omega_{c}+\omega_{b}/2$).

The wavelet transform of *u*(*x*, *t*) can be expressed as:

(19) $$WT(x,a,b)=\frac{1}{\sqrt{a}}\int_{-\infty }^{+\infty }u(x,t)\psi^{*}(\frac{t-b}{a})dt$$

where the superscript ‘*’ denotes a complex conjugate, *a* is the scale factor, and *b* is the time factor.

The impact signal can be expressed:

(20) $$u(x,t)=e^{-j(k_{1}x-\omega_{1}t)}+e^{-j(k_{2}x-\omega_{2}t)}$$

where *k*_1_ and *k*_2_ are the wave numbers. Introducing:

(21) $$\begin{array}{c} \Psi^{*}(a\omega )=\Psi^{*}(a\omega_{1})=\Psi^{*}(a\omega_{2})\\ \frac{k_{2}-k_{1}}{2}=\Delta k,\frac{\omega_{2}-\omega_{1}}{2}=\Delta \omega \\ \frac{k_{1}+k_{2}}{2}=k_{0},\frac{\omega_{1}+\omega_{2}}{2}=\omega_{0} \end{array}$$

The module values of the impact signal are acquired by the Shannon wavelet transform:

(22) $$|WT(x,a,b)|=\sqrt{2a}|\Psi (a\omega_{0})|\sqrt{1+\cos(\Delta \omega b-\Delta kx)}$$

When b=Δk/Δω=x/C, the module value is maximum, where *C* is the group of the velocity. Narrow band signal and module values can be obtained by Shannon wavelet transform. The time difference can be obtained by finding the peak times of the module values for wave velocities measurement.

## 3. Experiments

### 3.1. Wave Velocities Measurement

Because of the anisotropy of the composite material, the wave velocities of the elastic waves depend on the direction of wave propagation. According to Equation (5), the MUSIC algorithm requires the knowledge of the wave velocity. Hence, the wave velocities of all directions need to be measured.

**Figure 2:**
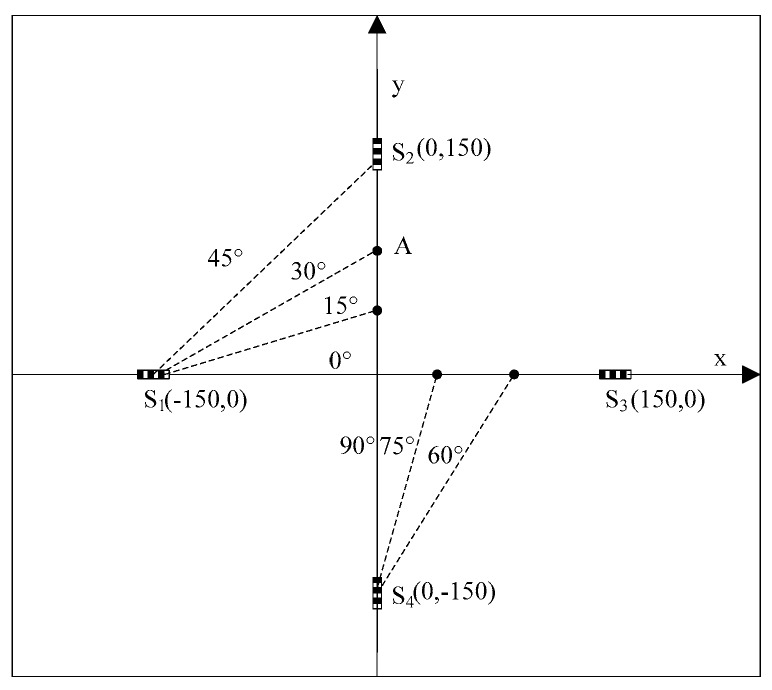
Measurement method of wave velocity.

A carbon fiber-reinforced polymer (CFRP) plate, the dimensions of which are 500 mm × 500 mm × 1.5 mm, was used for experiments. The ply sequence of the plate is [03/906/03]s. The four edges of the plate were clamped tightly by a metal frame. Four FBGs were glued on the plate, as shown in Figure 2. The coordinates of the FBGs are (150, 0), (0, 150), (−150, 0) and (0, −150), in the unit of mm. The impact events are generated by a steel ball with a diameter of 20 mm.

**Figure 3:**
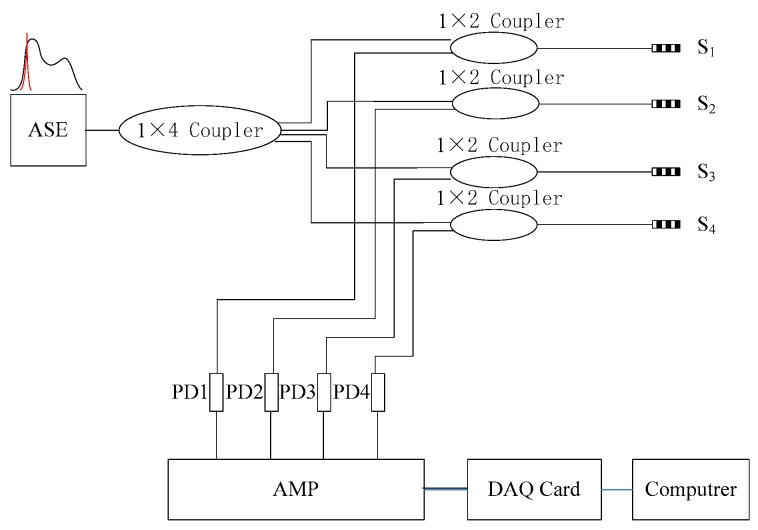
Wave velocities measurement system.

The signal demodulation system is composed of a white light source, fiber-optic couplers, photoelectric detectors and data acquisition equipment with a sampling frequency of 2.5 MHz, as shown in Figure 3. The wavelengths of four FBGs which constitute two sensor arrays are within the limits of 1542 ± 0.1 nm. The length of the FBG is 3 mm, and the reflectivity is 70%. The wavelength parameters of the FBGs all lie on the linear edge of the amplified spontaneous emission (ASE) source. The power demodulation method, which are composed of photoelectric detector (PD), amplifier (AMP), and data acquisition card (DAQ card), based on the edge filter principle [19], satisfies the need of acquiring high frequency signals. 

**Figure 4:**
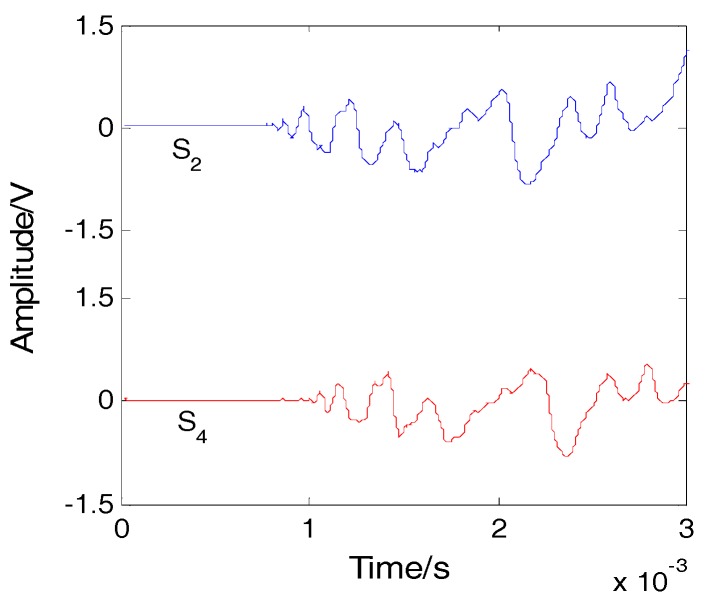
Impact signals.

**Figure 5:**
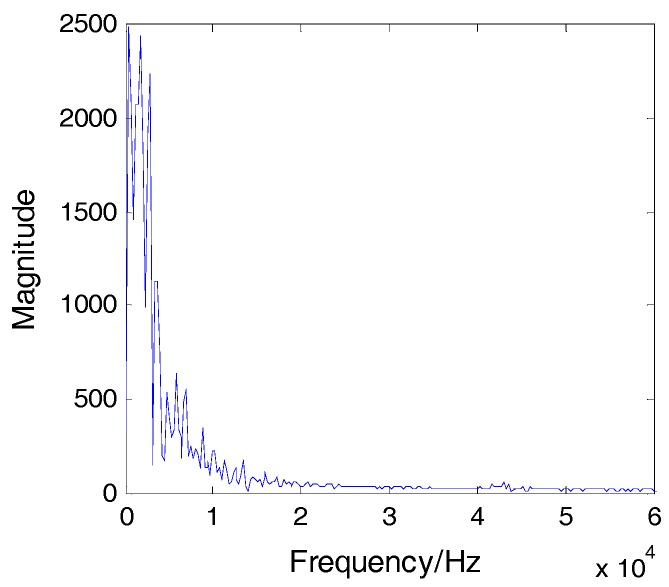
Frequency spectrum of S_2_.

**Figure 6:**
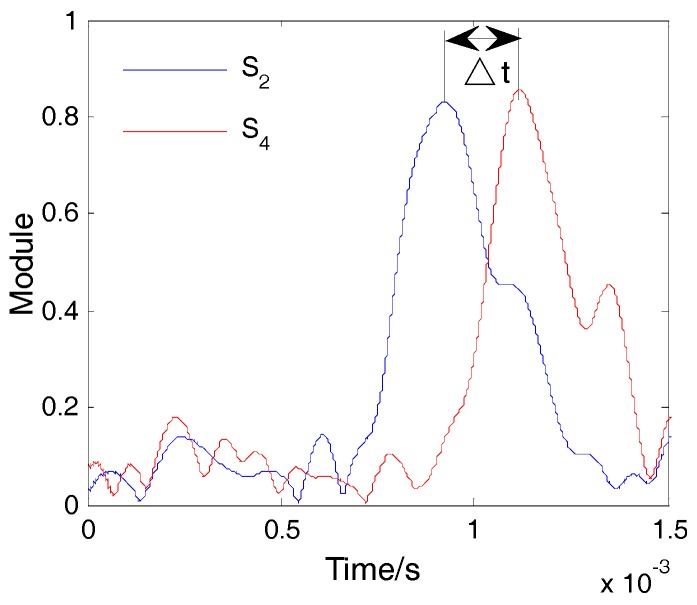
Time difference between S_2_ and S_4_.

**Figure 7:**
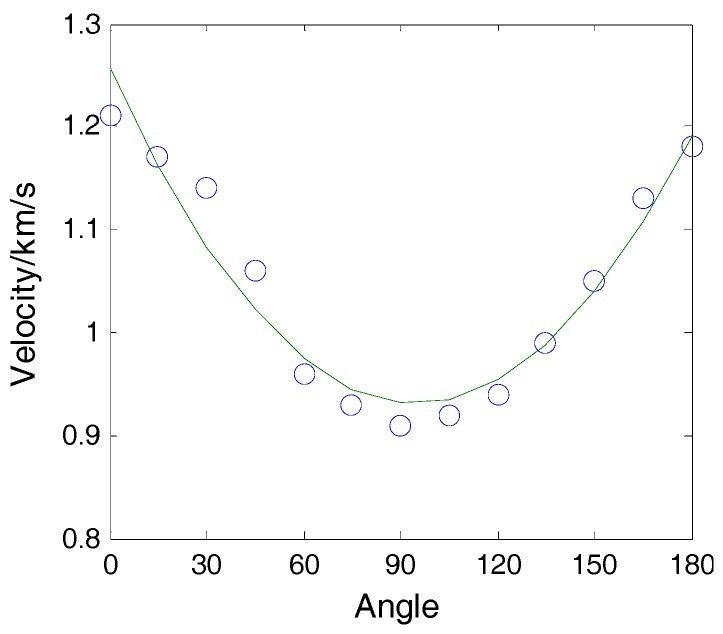
Wave velocities of different directions.

Impact was applied on the plate at location A, as shown in Figure 2. The included angle between line AS_1_ and the x-axis was 30°. The coordinate of point A was (0, 86.6 mm). The impact signals obtained by sensors S_2_ and S_4_ are shown in Figure 4 and the spectrum of the signal from S_2_ is shown in Figure 5. It is seen that the impact signal was a white band signal mainly located in the frequency band from 0 kHz to 50 kHz. The narrow band signals, whose central frequency is 40 kHz, were extracted by Shannon wavelet transform. The module values of the narrow band signals of S_2_ and S_4_ were calculated by Shannon wavelet transform, as shown in Figure 6. The first main peaks of the two modules were used to calculate the time difference between S_2_ and S_4_. According to the time difference and the difference between AS_2_ and AS_4_, the vertical wave velocity (90°) can be obtained. The time difference between S_1_ and S_4_ was calculated. According to the time difference, the vertical wave velocity and the difference between lines AS_1_ and AS_4_, the wave velocity was calculated at a 30° direction. Based on the above method, the wave velocities of all directions were obtained and the results are shown Figure 7.

### 3.2. Localization of Multiple Impacts

**Figure 8:**
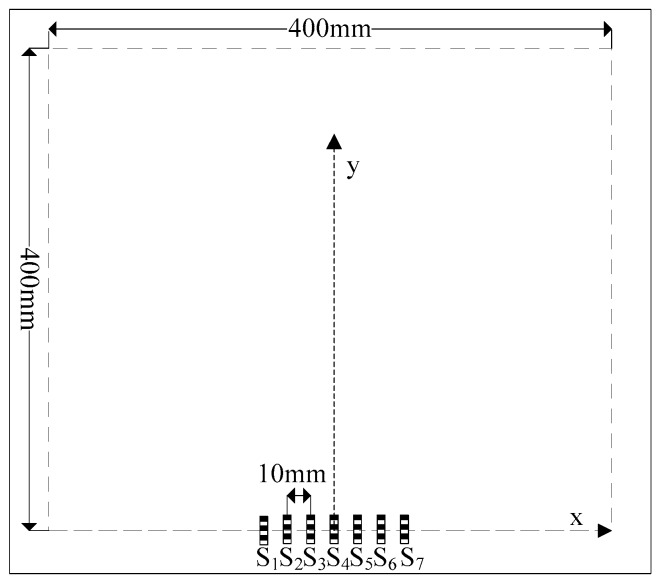
Localization experiment.

A sensor array consisting of seven FBGs with an equal spacing of 10 mm in a line was used for localization of impacts on a CFRP plate, as shown in Figure 8. The test area was a 400 mm × 400 mm square located at the center of the CFRP plate. The sensor S_4_ which is at (0, 0), was used as reference sensor. The signal demodulation system was similar to that shown in Figure 3. The testing area is divided into 160,000 (400 × 400) searching points.

**Figure 9:**
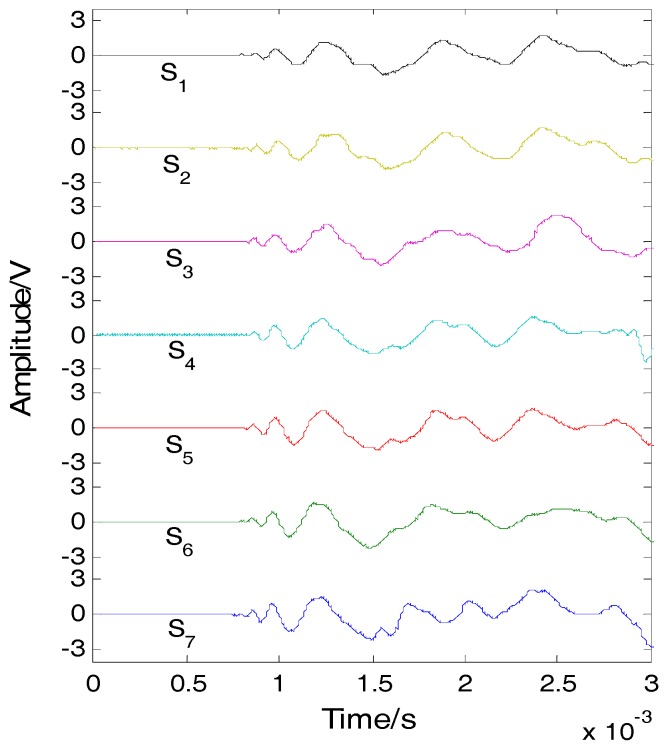
Impact signals of FBG array.

**Figure 10:**
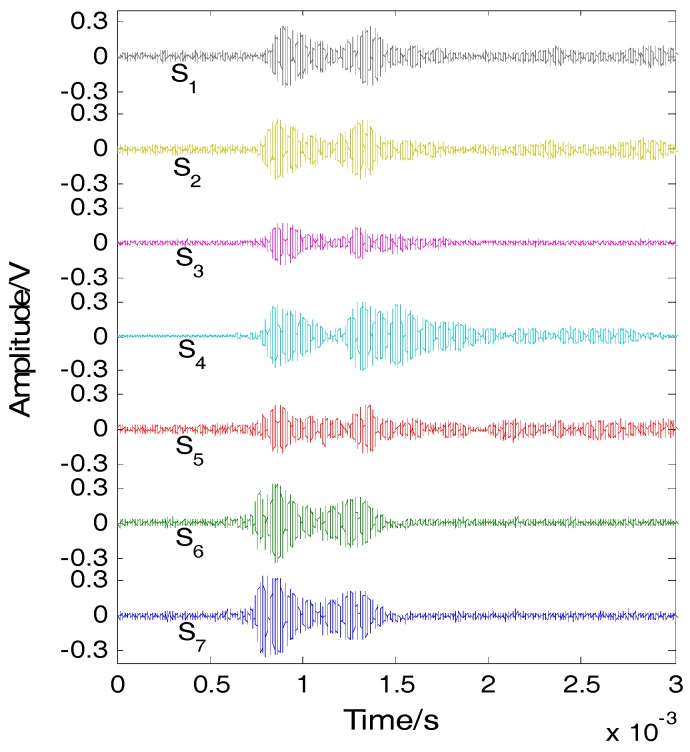
Narrow band signals.

Impacts were simultaneously applied on the plates using two steel balls at two different locations of (67 mm, 110 mm) and (177 mm, 319 mm). At the height of 200 mm above the CFRP plate, we used an electronic control valve device clip to hold the two balls, and through the control command, to get the valve to open as far as possible to make the two balls release at the same time in free fall. The signals detected by the FBG array are shown in Figure 9. The narrow band signals, whose center frequency is 40 kHz, were extracted, as shown Figure 10. According to the narrow band signals, the covariance matrix was calculated. Based on the Gerschgorin disc theorem, the GDE coefficients are GDE(1) = 63.6545, GDE(2) = 12.8813, GDE(3) = −5.0576, GDE(4) = −10.2319 and GDE(5) = −35.9862, respectively. Two out of the five GDE coefficients are positive; therefore the number of impacts was correctly found to be two. The eigenvectors of the covariance matrix were calculated. The eigenvectors of noise were extracted. The steering vector a(x, y) of each searching point was calculated.

**Figure 11:**
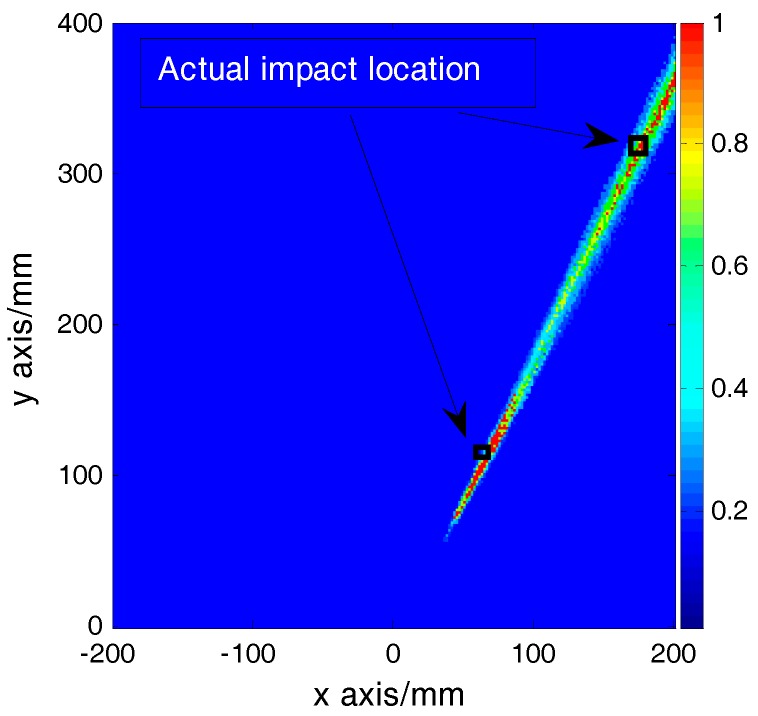
The localization spatial spectrum of (67,110) and (177,319).

Using the MUSIC algorithm, the spatial spectrum over the test area was calculated to obtain the locations of the impacts and the spatial spectrum is shown in Figure 11. The coordinates of the two peaks of the spatial spectrum are (69 mm, 108 mm) and (181 mm, 324 mm), respectively. To evaluate the accuracy of the results, an error function is defined as:

(23) $$\varepsilon =\sqrt{{\left(x_{actual}-x_{predicted}\right)}^{2}+{\left(y_{actual}-y_{predicted}\right)}^{2}}$$

where (*x_actual_*, *y_actual_*) is the actual coordinate of the impact source and (*x_predicted_*, *y_predicted_*) is the predicted coordinate of the impact source. According to Equation (23), the localization errors of dual-impacts are 2.8 mm and 6.4 mm, respectively.

**Figure 12:**
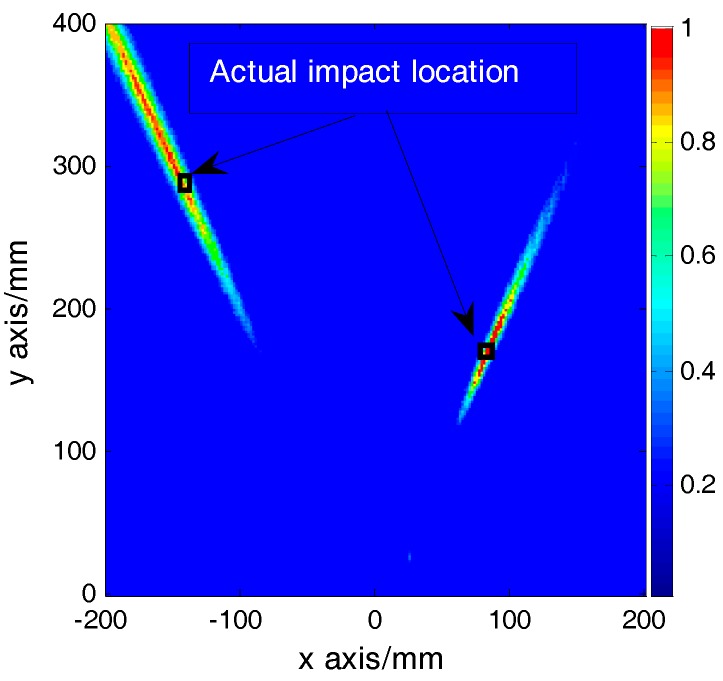
The localization spatial spectrum of (−149,289) and (89,172).

To further verify the performance of the impact detection system and the MUSIC algorithm, impact experiments were performed at another set of two randomly selected points. The coordinates of the impact points were (−149 mm, 289 mm) and (89 mm, 172 mm). The GDE coefficients were GDE(1) = 53.7112, GDE(2) = 9.7609, GDE(3) = −8.2491, GDE(4) = −11.6197 and GDE(5) = −21.9831, respectively. Again, from the number of positive GDE coefficients, the number of impacts was correctly found to be two. The localization spatial spectrum is shown in Figure 12. The localization errors are 8.6 mm and 3.6 mm. Another five sets of dual-impact localization experiments were carried out and the results are summarized in Table 1 and Table 2. The GDE coefficients are shown in Table 1. According to the number of GDE coefficients larger than zero, the numbers of five dual-impacts are all two. The localization results are shown in Table 2. We can find that the maximum error and average error are 9.2 mm and 7.4 mm, respectively. The results confirm that the proposed algorithm can be applied for multi-impact localization of composite structures.

## 4. Conclusions

In this paper, a multi-impact localization system based on a FBG sensor array and the MUSIC algorithm was investigated. The signals from multiple impacts were detected by the FBG array and Shannon wavelet transform was used to extract the narrow band signals of the impact signals. The Gerschgorin disc theorem was used for estimating the number of impacts. The MUSIC algorithm was employed to obtain the coordinates of multiple impacts. The system and algorithm were verified on a 500 mm × 500 mm × 1.5 mm CFRP plate. The results showed that the maximum error and average error of multi-impact localization are 9.2 mm and 7.4 mm, respectively. The designed system and algorithm can achieve the CFRP structural multi-damage identification reliably.

## Figures and Tables

**Table 1:** GDE coefficients.

Impact Event	GDE Coefficients				
	GDE(1)	GDE(2)	GDE(3)	GDE(4)	GDE(5)
1	38.1901	10.8721	−5.6729	−11.0569	−20.6672
2	29.1176	8.2952	−4.8572	−10.2598	−20.2149
3	43.5331	13.6286	−8.7215	−15.6943	−23.2517
4	51.4519	16.8921	−10.3996	−18.9185	−26.2364
5	39.2379	11.9012	−8.1339	−14.8913	−22.7269

**Table 2:** Experimental results.

Number	Actual Coordinate (mm)		Predicted Coordinate (mm)		Error (mm)	
1	(−171,58)	(−127,138)	(−176,65)	(−133,144)	8.6	8.4
2	(−119,216)	(57,268)	(−112,210)	(53,260)	9.2	8.9
3	(−52,157)	(132,96)	(−59,156)	(137,98)	7.7	5.3
4	(86,117)	(92,279)	(89,121)	(96,283)	5	5.6
5	(159,107)	(78,193)	(163,111)	(83,198)	8	7
